# In Vivo and In Vitro Antioxidant Effects of *Arthrospira platensis* Polysaccharide Component 1 (PAP-1)

**DOI:** 10.3390/antiox14111358

**Published:** 2025-11-13

**Authors:** Haifeng Yuan, Yuheng Wei, Zhaoyuan He, Xinrui Wang, Xiaoli Yu, Qiuhua Wang, Meiling Yu, Tingjun Hu

**Affiliations:** College of Animal Science and Technology, Guangxi University, Nanning 530004, China; 2318302040@st.gxu.edu.cn (H.Y.); weiyuheng@st.gxu.edu.cn (Y.W.); z294he@uwaterloo.ca (Z.H.); 2018393054@st.gxu.edu.cn (X.W.); 2318393087@st.gxu.edu.cn (X.Y.); qiuhuawang@gxu.edu.cn (Q.W.); yumeiling@gxu.edu.cn (M.Y.)

**Keywords:** *Arthrospira platensis*, polysaccharide component (PAP-1), antioxidant behaviour, oxidative stress, RNA-seq, in vivo study, cellular study

## Abstract

*Arthrospira platensis* polysaccharide component 1 (PAP-1), a purified polysaccharide monomer isolated from *Arthrospira platensis*, exhibits pronounced antioxidant activity. To investigate the in vivo and in vitro regulatory effects of PAP-1 on antioxidant enzyme activities and inflammatory mediators in mice and RAW264.7 cells, the mice were administered PAP-1 by gavage, and the cells were cultured with PAP-1. Subsequently, serum, lung, spleen, and thymus tissues from mice, as well as the cultured RAW264.7 cells, were collected for analysis using RNA sequencing, commercial assay kits, immunohistochemistry, RT-qPCR, and Western blotting. The results demonstrated that PAP-1 significantly reduced the levels of oxidative stress-related indicators (NO, iNOS, MDA, MPO, and XOD), while markedly enhancing the activities of antioxidant enzymes (SOD, CAT, and GSH-Px) (*p* < 0.05), a trend consistently observed in both in vivo and in vitro experiments. Furthermore, PAP-1 upregulated the expression of key antioxidant genes and proteins, including HO-1, NQO1, GCLM, p62, Prdx1, and SLC7A11. Collectively, these findings indicate that PAP-1 exerts regulatory antioxidant effects in mice and RAW264.7 cells by enhancing antioxidant enzyme activity and suppressing oxidative stress responses, underscoring its potential as a natural antioxidant agent.

## 1. Introduction

Oxidative stress is a pathological state characterized by an imbalance between the generation of reactive oxygen species (ROS) during metabolic processes and the capacity of the antioxidant defense system [1]. The excessive accumulation of ROS and high concentrations of oxygen radicals can not only compromise the structural integrity of cells and downregulate intracellular reductive capacity but also regulate cellular processes such as proliferation, apoptosis, and gene expression, ultimately activating downstream signaling pathways that contribute to the onset and progression of various diseases [2]. In the event of an imbalance in the redox homeostasis of the organism or during specific metabolic redox processes, the excessive generation of free radicals can, on one hand, participate in the regulation of the intricate interplay between the enzymatic reaction systems and the non-enzymatic defense mechanisms. On the other hand, these radicals can selectively target and damage critical antioxidant molecules, ultimately impairing the physiological functions of the organism [3]. Therefore, repairing and alleviating systemic damage or functional dysregulation induced by ROS, and promoting the detoxification roles of peroxidation products (PPs), has become a central focus in the field of antioxidation. However, research on redox reaction systems continues to face numerous unresolved challenges.

ROS, as pivotal secondary messengers in cellular signal transduction, participate in the metabolic processes of various cytokines and regulate vital cellular functions. Within the cellular environment, the clearance of ROS is primarily mediated by a cooperative action involving GSH-Px, SOD, and CAT [4]. GSH-Px, as a crucial hydrogen peroxide (H_2_O_2_) degrading enzyme, relies on glutathione (GSH) as its substrate to mediate the decomposition of H_2_O_2_, thereby maintaining the structural integrity and functional homeostasis of the cell membrane [5]. SOD, as a key enzyme in regulating the cytotoxic effects of superoxide anions, not only prevents and mitigates cell damage induced by extracellular superoxide through pretreatment of cells or tissues, but also enhances the cell’s resistance and tolerance to both oxidative and non-oxidative dual stresses [6]. CAT catalyzes redox reactions of substrates, efficiently eliminating various ROS; it enhances the body’s capacity to suppress hydroxyl radicals and superoxide anions, thereby exerting potent antioxidant effects [7]. Moreover, the catalytic activity of CAT significantly reduces the levels of MDA [8]. Redox enzymes primarily encompass key members such as XOD, CAT, SOD, GSH-Px, and MPO. Following years of systematic scientific investigation, these enzymes have established a robust foundation in both theoretical and experimental research, and they are now regarded as core indicators for evaluating the functional status of the antioxidant defense system.

The screening and extraction of natural antioxidant compounds from plant-based resources for applications in food processing and pharmaceutical development have long been a focal point of interest across both academia and industry. As an environmentally friendly algal species, *Arthrospira platensis* is characterized by its rapid growth, low nutrient and water requirements [9], and its capacity for large-scale cultivation in seawater following domestication. At present, China ranks first globally in *Arthrospira platensis* production [10,11]. Due to its natural origin and abundant cultivation reserves, *Arthrospira platensis* has attracted widespread attention from researchers. Modern pharmacological studies have revealed that *Arthrospira platensis* polysaccharides possess notable pharmacological activities, including anti-inflammatory [12] and antioxidant [13] properties. Polysaccharides extracted from *Arthrospira platensis* not only exhibit strong scavenging activity against hydroxyl radicals and DPPH free radicals [14], but also demonstrate a protective effect against hearing loss induced by oxidative stress from ototoxic drugs [15]. The *Arthrospira platensis* polysaccharide complex (SPC) restores mitochondrial dysfunction induced by ROS in senescent fibroblasts by upregulating superoxide dismutase 2 (SOD2), thereby enhancing the clearance of superoxide radicals [16]. The above studies indicate that *Arthrospira platensis* polysaccharides serve as effective antioxidants.

In our previous studies [14], structural analysis was performed on PAP-1, a purified polysaccharide isolated from *Arthrospira platensis*, and in vitro free radical scavenging assays revealed its potent antioxidant activity. Furthermore, PAP-1 was shown to mitigate IL-17-mediated inflammation via the ceRNA mechanism by targeting receptors in the IL-17 signaling pathway. While these findings highlight its anti-inflammatory and antioxidant potential, a comprehensive understanding of its antioxidant effects remains to be established. This study therefore aims to fully investigate the antioxidant properties of PAP-1 in vitro and in vivo, providing a scientific basis for developing novel antioxidant therapeutics.

## 2. Materials and Methods

### 2.1. Reagents and Solution Preparation

All reagents and preparation solutions are shown in Supplementary Materials.

### 2.2. Animal and RAW264.7 Cell Grouping and Treatment

Animal and RAW264.7 cell grouping and treatment are shown in Supplementary Materials.

### 2.3. Preparation of Samples for RNA-Seq

Two experimental groups were established for RNA-Seq: the PAP-1 treatment group (200 μg/mL) and the cell control group, each with three biological replicates. After RAW264.7 cells reached appropriate confluency, the culture supernatant was discarded, and cells were washed three times with PBS. The PAP-1 group was treated with 200 μg/mL of freshly prepared, sterile-filtered PAP-1 solution, while the control group received an equal volume of complete culture medium. All plates were incubated for 12 h at 37 °C in a 5% CO_2_ humidified incubator. Following incubation, the supernatant was removed, and cells were gently washed with PBS. Subsequently, 1 mL of Trizol reagent was added to each well to lyse the cells. The lysates were collected and stored at −80 °C until total RNA extraction for RNA-Seq.

### 2.4. RNA-Seq Sample Relationships and GO/KEGG Analysis

The relationships among RNA-Seq samples were assessed using principal component analysis (PCA) scatter plots and correlation heatmaps to evaluate the reproducibility and variability among the three biological replicates within each group. Differentially expressed mRNAs between the PAP-1 group (200 μg/mL) and the control group were identified based on the criteria of *p* < 0.05 and FC > 1.5. Subsequent Gene Ontology (GO) and Kyoto Encyclopedia of Genes and Genomes (KEGG) enrichment analyses were performed to identify the most significantly enriched terms and signaling pathways.

### 2.5. Calculation of Mouse Organ Index

Following euthanasia, the spleen, thymus, and lungs were carefully excised and gently blotted with filter paper to remove surface moisture. The respective organ indices were calculated by using the formula: Organ Index = Organ Weight (mg)/Body Weight (g) [17].

### 2.6. Preparation of Histopathological Sections from Murine Tissues

Following experimental euthanasia, the lungs, spleen, and thymus were promptly harvested for pathological evaluation. Tissues were fixed in 4% paraformaldehyde at room temperature for 24 h, followed by routine dehydration and paraffin embedding. Sections were then cut at a thickness of 5 μm and subjected to hematoxylin and eosin (H&E) staining to assess histopathological alterations [18].

### 2.7. Immunohistochemical Analysis of Murine Tissues

Formalin-fixed, paraffin-embedded (FFPE) [19] spleen and thymus tissues from mice were sectioned at a thickness of 3.5~4 μm. Antigen retrieval was performed by immersing the sections in 0.01 M sodium citrate buffer (pH 6.0). Following a 20 min blocking step at room temperature, the sections were incubated with 1:100 diluted biotinylated goat anti-mouse IgG primary antibody and streptavidin-POD at 20~37 °C for 30 min. After development with DAB chromogen and light hematoxylin counterstaining, the sections were dehydrated, cleared, and mounted for microscopic examination. DAB staining intensity was quantified using ImageJ software (version 1.54p). Immunohistochemical results were interpreted based on the intensity of cytoplasmic or membrane staining and categorized as negative, weakly positive, moderately positive, or strongly positive. The percentage of positively stained cells was used as an index of expression strength.

### 2.8. Western Blot Analysis

Total protein was extracted from tissues and cells strictly according to the instructions of the corresponding protein extraction kits, with the nuclear protein extraction step optimized based on the acid-based method. Protein concentrations were determined using the BCA Protein Assay Kit (Suzhou NewSaier Biotech Co., Ltd., Suzhou, China) following the manufacturer’s protocol. Equal amounts of protein were separated via SDS-PAGE using a discontinuous gel system and subsequently transferred onto PVDF membranes. The membranes were incubated overnight at 4 °C with primary antibodies, washed three times with TBST, and then incubated with BSA-diluted secondary antibodies at room temperature for 2 h [20]. Protein expression levels were quantified by measuring the grayscale intensity of target bands using ImageJ-Win software. The relative expression of each target protein was calculated as the ratio of the grayscale intensity of the target band to that of the corresponding internal control.

### 2.9. RT-qPCR Analysis

Parameter for RT-qPCR analysis are shown in Supplementary Materials and Table S2.

### 2.10. Data Processing and Statistical Analysis

In vitro experiment (RAW264.7 cells): Three independent replicates were conducted for each group (*n* = 3); In vivo animal experiment (mice): Each group consisted of ten samples (*n* = 10). Data analysis was conducted using GraphPad Prism 9.4.0 employing ordinary one-way ANOVA. Results are presented as mean ± standard deviation (Mean ± SD). Given the limited number of experimental groups and the satisfaction of homogeneity of variance, intergroup comparisons were conducted using the LSD-t test. The consistency observed across multiple independent experiments further validated the reliability of the results. Differences were considered statistically significant at *p* < 0.05 and highly significant at *p* < 0.01.

## 3. Results

### 3.1. Transcriptome Sequencing Sample Correlation and Differential Gene Analysis

**Figure 1:**
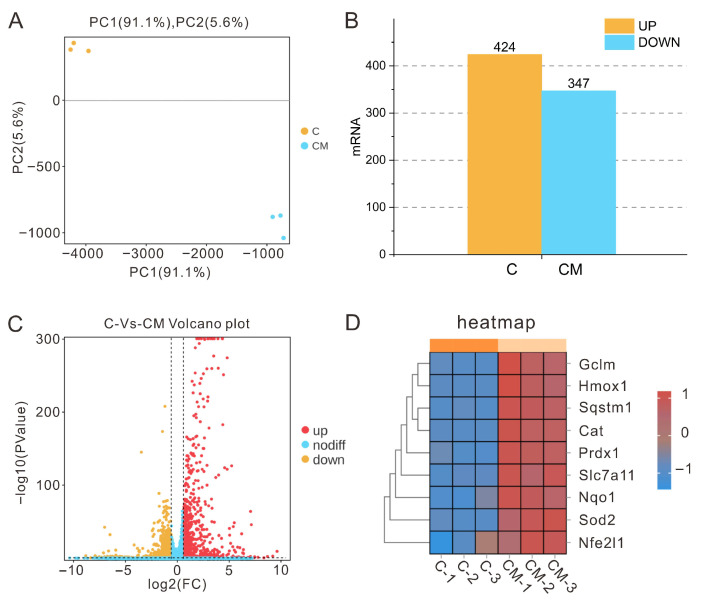
Transcriptomic sequencing sample relationships and differential gene analysis (means ± SD, *n* = 3). (**A**) PCA scatter plot (yellow dots represent the cell treatment group, blue dots denote the 200 μg/mL PAP-1 group, with *n* = 3 biological replicates per group). (**B**) Bar chart illustrating the number of differentially expressed genes. (**C**) Volcano plot of differential gene expression. (**D**) Heatmap of differential expression of key antioxidant-related genes.

Transcriptome sequencing revealed that PAP-1 exerted a pronounced impact on cellular transcriptional profiles. Principal component analysis (PCA) scatter plots (Figure 1A) demonstrated a clear spatial separation between the control group and the 200 μg/mL PAP-1 treatment group, with high intra-group consistency, indicating reliable experimental data and significant inter-group differences. Differential gene expression analysis (*p* < 0.05, FC > 1.5) identified 424 significantly upregulated and 347 significantly downregulated mRNAs (Figure 1B,C). Further examination showed that, compared with the control group, numerous antioxidant-related genes including HO-1, NQO1, GCLM, p62, Prdx1, SLC7A11, CAT, SOD2, and Nrf1 were markedly upregulated following PAP-1 treatment (Figure 1D). These results suggest that PAP-1 may enhance cellular resilience to oxidative stress by activating endogenous antioxidant defense pathways.

### 3.2. Differential GO/KEGG Analysis

To further elucidate the biological functions of differentially expressed genes following PAP-1 treatment, GO and KEGG enrichment analyses were performed (Figure S1). The results revealed that these genes were predominantly enriched in biological processes associated with the regulation of oxidative stress, immune responses, and signal transduction. Notably, they were significantly involved in several canonical antioxidant and inflammation-related signaling pathways, including the FoxO, IL-17, NF-κB, and Toll-like receptor pathways, suggesting that PAP-1 may exert its antioxidative and anti-inflammatory effects through the coordinated modulation of multiple signaling networks.

### 3.3. In Vivo Antioxidant Effects of PAP-1

#### 3.3.1. Effects of *Arthrospira platensis* Polysaccharides on Redox-Related Biomarkers in Mice

**Figure 2:**
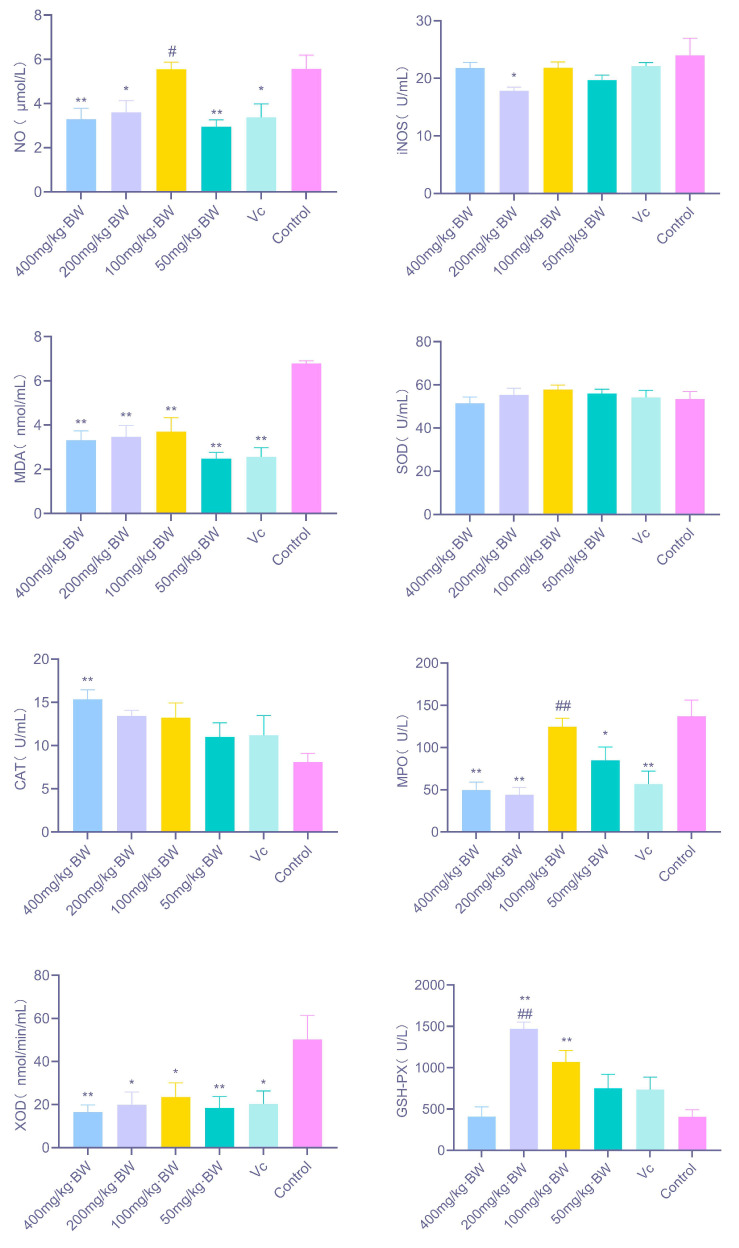
Effects of PAP-1 on redox-related factors in mice (means ± SD, *n* = 10). The figure illustrates changes in NO, iNOS, MDA, CAT, MPO, XOD, and GSH-Px under different dosage treatments. Statistical significance is denoted as follows: * *p* < 0.05 and ** *p* < 0.01 vs. control group; # *p* < 0.05 and ## *p* < 0.01 vs. Vc group; same notation applies hereafter.

As illustrated in Figure 2, PAP-1 exhibited a pronounced regulatory effect on redox homeostasis in mice across varying dosages. Specifically, treatment with 400 mg/kg·BW and 50 mg/kg·BW significantly reduced systemic NO levels (*p* < 0.01), while 200 mg/kg·BW also elicited a notable decrease (*p* < 0.05). In the case of iNOS, only the 200 mg/kg·BW dosage induced a significant downregulation (*p* < 0.05). All tested dosages led to a highly significant reduction in MDA content (*p* < 0.01). Moreover, 400 mg/kg·BW markedly enhanced CAT activity (*p* < 0.01). Regarding MPO, both 400 mg/kg·BW and 200 mg/kg·BW induced a highly significant decrease in activity (*p* < 0.01), whereas 50 mg/kg·BW produced a significant decline (*p* < 0.05). For XOD, significant downregulation was observed at 400 mg/kg·BW and 50 mg/kg·BW (*p* < 0.01), with 200 mg/kg·BW and 100 mg/kg·BW also demonstrating significant reductions (*p* < 0.05). In addition, both 200 mg/kg·BW and 100 mg/kg·BW markedly increased GSH-Px activity (*p* < 0.01), suggesting that PAP-1 may preserve systemic redox equilibrium by simultaneously enhancing endogenous antioxidant enzyme activity and suppressing pro-oxidant enzyme production.

#### 3.3.2. Results of Organ Index and Histopathological Analysis in Mice

**Figure 3:**
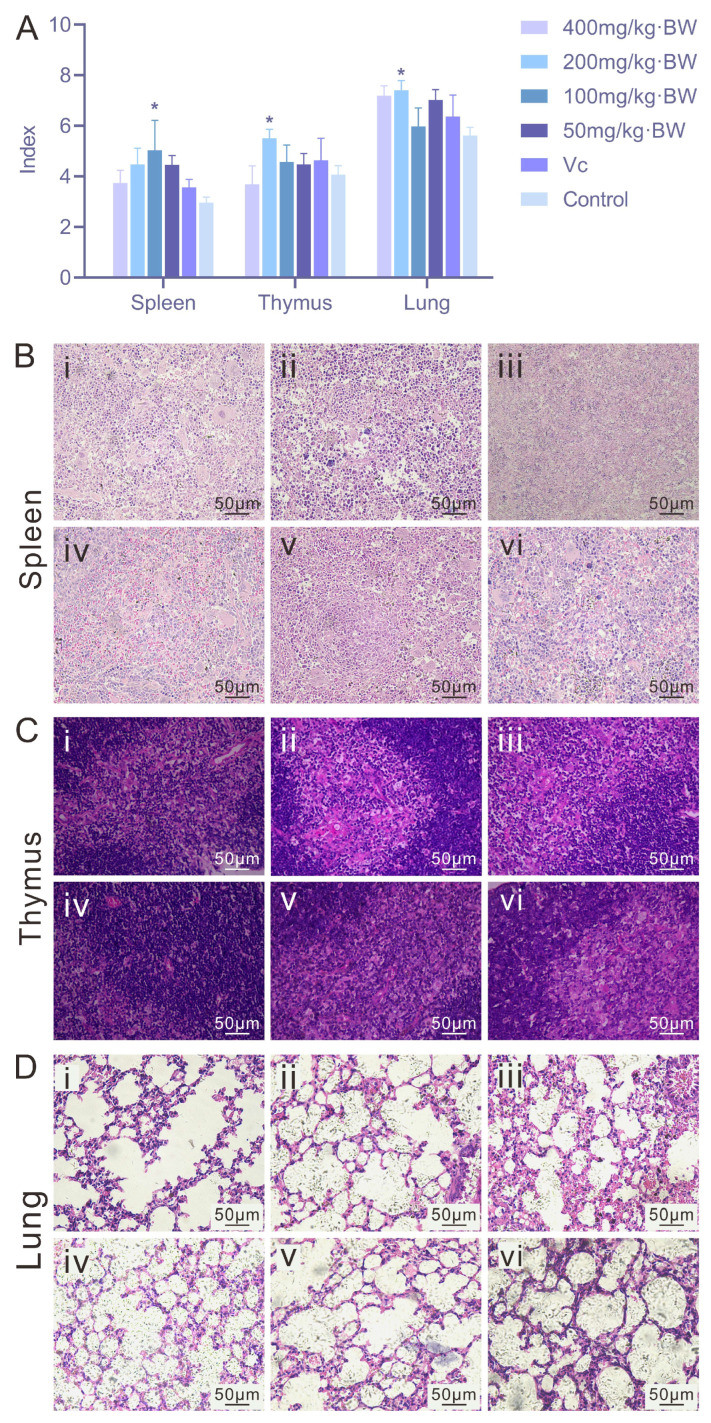
Effects of PAP-1 on organ indices and histopathology in mice (means ± SD, *n* = 10). (**A**) Comparison of spleen, thymus, and lung indices. (**B**) Histological changes in the spleen (HE staining, ×400). (**C**) Histological changes in the thymus (HE staining, ×400). (**D**) Histological changes in the lung (HE staining, ×400). i: 400 mg/kg·BW PAP-1 group; ii: 200 mg/kg·BW PAP-1 group; iii: 100 mg/kg·BW PAP-1 group; iv: 50 mg/kg·BW PAP-1 group; v: Vc group; vi: blank control group (The same interpretation applies to Figure 4 and Figure 5).

As shown in Figure 3A, PAP-1 enhanced the development of immune organs at certain dosages compared with the blank control group. Specifically, mice administered 200 mg/kg·BW exhibited significantly increased thymus and lung indices (*p* < 0.05), while the 100 mg/kg·BW dosage significantly elevated the spleen index (*p* < 0.05). In spleen sections (Figure 3B), all PAP-1 treatment groups displayed evident proliferation of the white pulp, high lymphocyte density, well-defined marginal zones, and abundant macrophages and plasma cells, suggesting that PAP-1 may promote splenic immune responsiveness. In thymus sections (Figure 3C), PAP-1 treatment (400, 200, 100, and 50 mg/kg·BW) resulted in a distinct cortex-medulla boundary, tightly arranged cells, an increased number of thymic lymphocytes, and more intact cellular architecture compared with the blank control. In lung tissue sections (Figure 3D), both PAP-1 groups and the Vc positive control group demonstrated reduced alveolar septal thickness, enlarged alveolar diameters, narrowed peribronchiolar spaces, and amelioration of ciliary shedding, with no evident inflammatory cell infiltration in any group.

### 3.4. Immunohistochemical Analysis

#### 3.4.1. Expression of NQO1 and HO-1 in Murine Splenic Tissue

**Figure 4:**
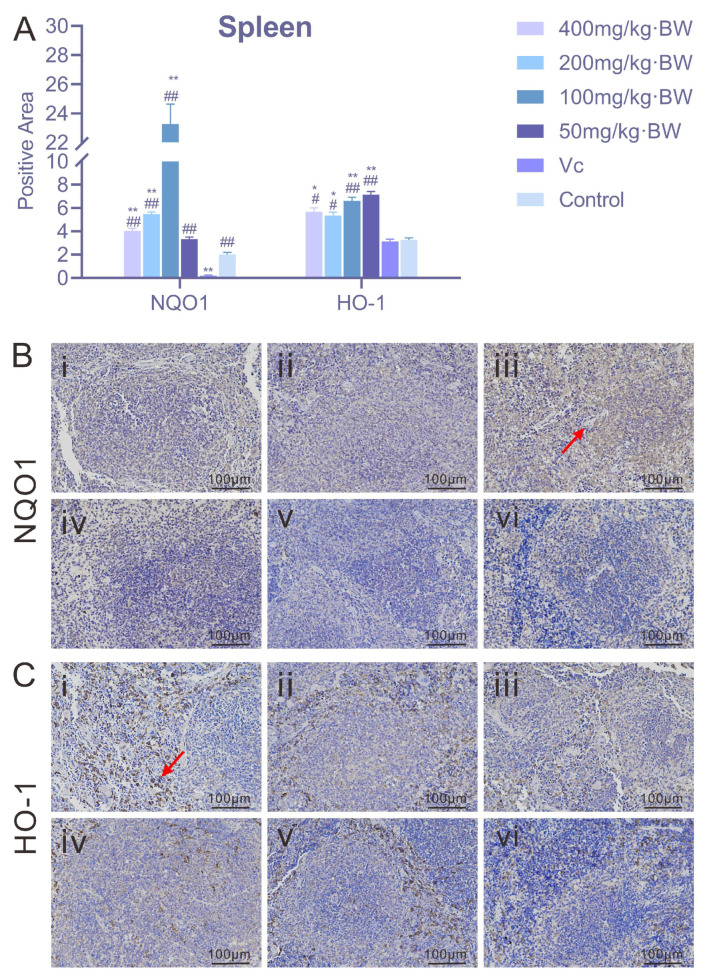
Effects of PAP-1 on the expression of NQO1 and HO-1 in mouse spleen (means ± SD, *n* = 10). (**A**) Positive rates of NQO1 and HO-1 in the spleen (%Area). (**B**) Immunohistochemical staining of NQO1 (IHC, ×200), with brownish-yellow granules primarily localized in the cytoplasm (arrows indicate positive expression). (**C**) Immunohistochemical staining of HO-1 (IHC, ×200), with brownish deposits predominantly localized in the nuclei (arrows indicate positive expression).

As illustrated in Figure 4, PAP-1 exerted a regulatory effect on the expression levels of NQO1 and HO-1 in the spleen of a mouse. For NQO1, the positive rate in the 200 mg/kg·BW and 100 mg/kg·BW treatment groups reached ≥5%, exhibiting a pronounced upregulation; by contrast, the positive rate in the 400 mg/kg·BW and 50 mg/kg·BW groups fell below 5%. The activation of NQO1 was particularly notable at moderate doses of PAP-1, suggesting the presence of a “nonlinear relationship.” Regarding HO-1 expression, all PAP-1 treatment groups (400, 200, 100, and 50 mg/kg·BW) as well as the vitamin C control group displayed positive rates ≥5%. Immunohistochemical staining of spleen tissues revealed distinct brownish deposits of HO-1 predominantly localized within the nuclei, indicating a broadly enhanced expression pattern induced by PAP-1. The sustained activation of HO-1 may confer a stable cytoprotective effect on the organism.

#### 3.4.2. Expression of NQO1 and HO-1 in Murine Thymic Tissue

**Figure 5:**
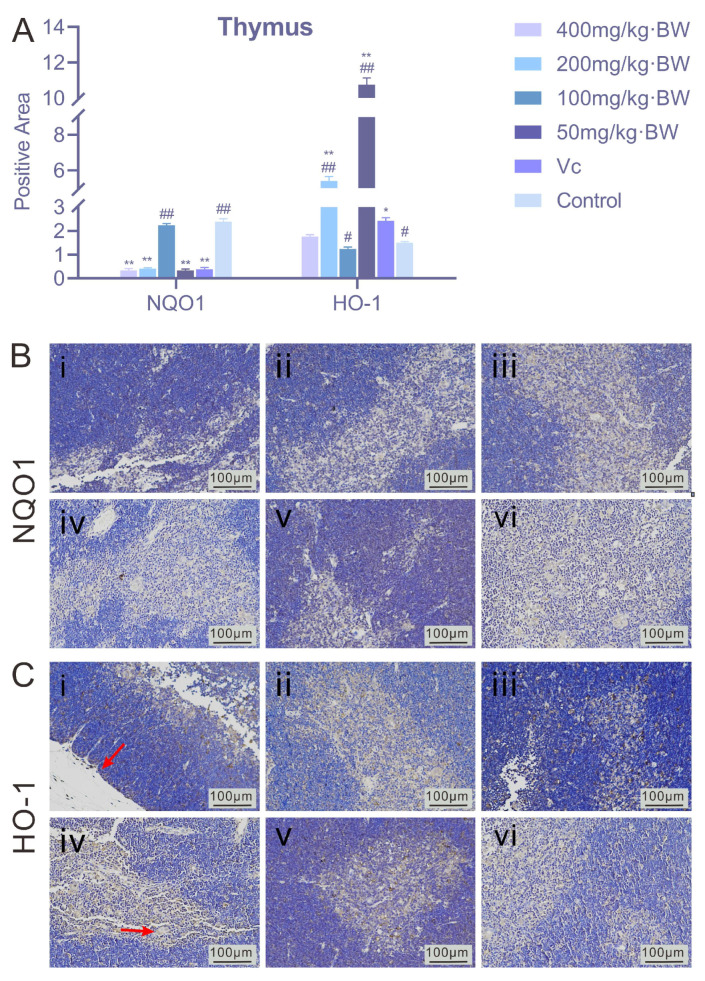
Effects of PAP-1 on the expression of NQO1 and HO-1 in mouse thymus (means ± SD, *n* = 10). (**A**) Positive rates of NQO1 and HO-1 in the thymus (%Area). (**B**) Immunohistochemical staining of NQO1 (IHC, ×200). (**C**) Immunohistochemical staining of HO-1 (IHC, ×200), showing brownish deposits predominantly in the thymic medulla (arrows indicate HO-1 positive expression).

As shown in Figure 5, PAP-1 did not produce a pronounced effect on NQO1 expression in the murine thymus. Relative to the blank control, the NQO1-positive area in all treatment groups (400, 200, 100, and 50 mg/kg·BW) remained below 5%, indicating a limited induction of NQO1 in the thymus. This phenomenon may be attributed to the intrinsically low basal expression of NQO1 in this organ or to distinct local requirements for antioxidative defense. For HO-1, however, the expression patterns varied across doses. The 200 mg/kg·BW and 50 mg/kg·BW PAP-1 treatment groups exhibited positive rates ≥5%, whereas the 400 mg/kg·BW and 100 mg/kg·BW groups remained below 5%. Immunohistochemical staining revealed that HO-1 granules were primarily deposited in the medullary region of the thymus, with sparse expression observed in the capsule. These findings suggest that PAP-1 induced HO-1 expression in a dose-dependent yet nonlinear manner, where moderate and low doses were more effective in triggering HO-1 upregulation than higher doses.

### 3.5. In Vitro Antioxidant Activity of PAP-1

#### 3.5.1. Effects of PAP-1 on Redox-Related Factor Levels in RAW264.7 Cells

**Figure 6:**
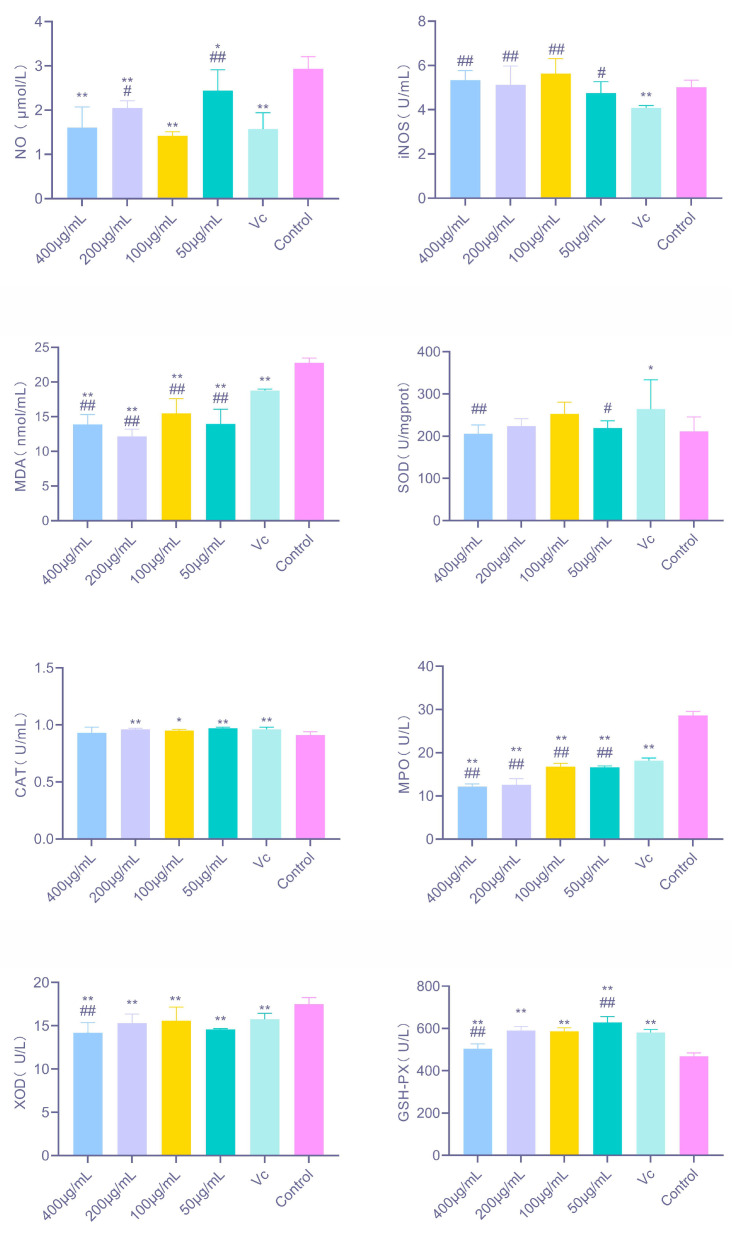
Effects of PAP-1 on redox-related factors in RAW264.7 cells (means ± SD, *n* = 3). Shown are the changes in NO, iNOS, MDA, CAT, MPO, XOD, and GSH-Px under different treatment concentrations.

As shown in Figure 6, PAP-1 exerted a significant regulatory effect on the redox status of RAW264.7 macrophages. Compared with the control group, PAP-1 markedly reduced NO levels at concentrations of 400, 200, and 100 μg/mL (*p* < 0.01), and significantly decreased MDA levels at all tested concentrations (*p* < 0.01). CAT activity was significantly elevated at 200 μg/mL and 50 μg/mL (*p* < 0.01), with a notable increase also observed at 100 μg/mL (*p* < 0.05). In addition, PAP-1 treatment at all four concentrations significantly enhanced GSH-Px activity (*p* < 0.01). Furthermore, PAP-1 consistently inhibited MPO and XOD activities across all concentrations (*p* < 0.01), suggesting that it not only blocks excessive ROS production induced by overactivation of oxidases, but may also indirectly alleviate cellular damage by downregulating inflammation-related oxidases such as MPO.

#### 3.5.2. mRNA Expression Levels of Antioxidant-Related Factors in RAW264.7 Cells Under the Influence of PAP-1

**Figure 7:**
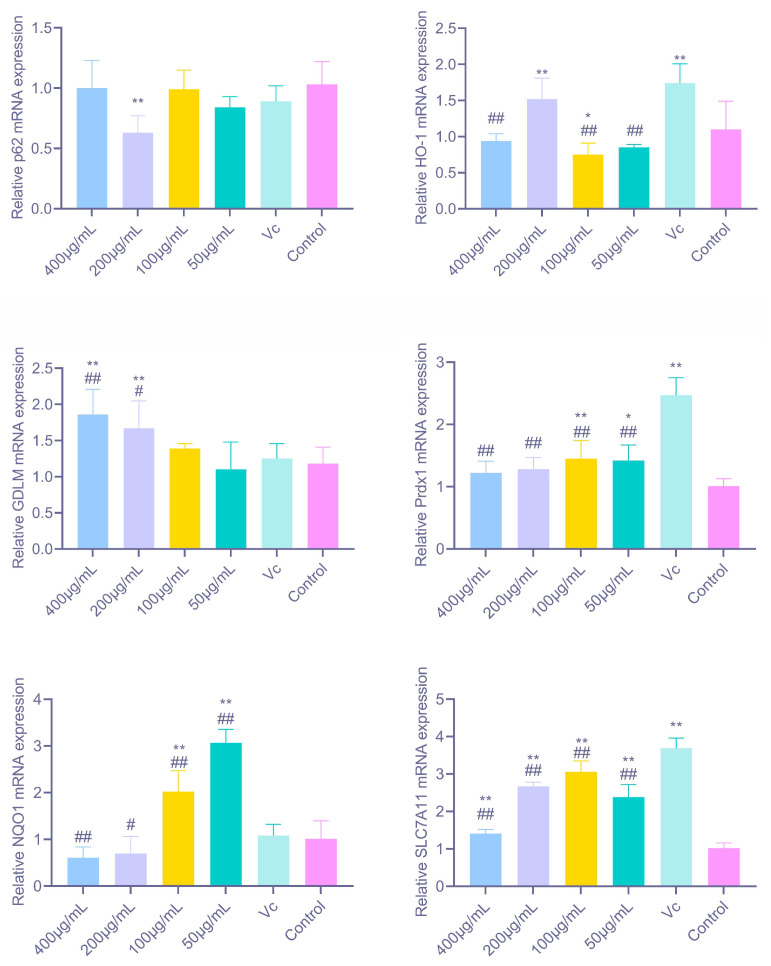
Effects of PAP-1 on the mRNA expression of antioxidant-related factors in RAW264.7 cells (means ± SD, *n* = 10). Genes examined include HO-1, NQO1, GCLM, p62, Prdx1, and SLC7A11.

As shown in Figure 7, PAP-1 significantly modulated the gene expression of multiple antioxidant-related factors in RAW264.7 cells. Among the canonical Nrf2 target genes, 200 μg/mL PAP-1 markedly upregulated HO-1 mRNA expression (*p* < 0.01); NQO1 expression was strongly elevated at 100 μg/mL and 50 μg/mL (*p* < 0.01); while GCLM expression was significantly enhanced by 400 μg/mL and 200 μg/mL PAP-1 (*p* < 0.01). These findings indicate that PAP-1 activates different branches of the Nrf2 signaling pathway in a dose-dependent manner, thereby initiating diverse antioxidant defense mechanisms. For other antioxidant and stress-related factors, 200 μg/mL PAP-1 significantly downregulated p62 mRNA expression (*p* < 0.01). Prdx1 expression was markedly increased at 100 μg/mL (*p* < 0.01) and significantly elevated at 50 μg/mL (*p* < 0.05), suggesting that PAP-1 enhances cellular capacity for hydrogen peroxide clearance. Moreover, SLC7A11 expression was strongly upregulated at all tested concentrations (*p* < 0.01).

#### 3.5.3. Protein Expression Levels of Antioxidant-Related Factors in RAW264.7 Cells Under the Influence of PAP-1

**Figure 8:**
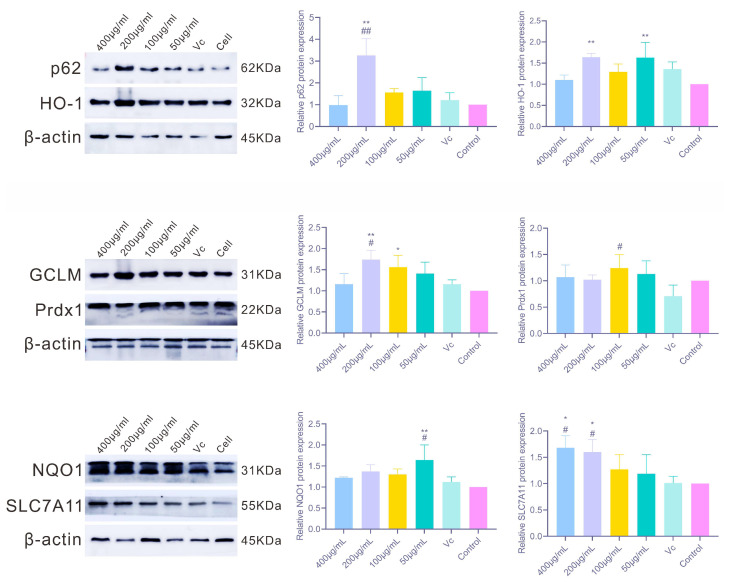
Effects of PAP-1 on the protein expression of antioxidant-related factors in RAW264.7 cells (means ± SD, *n* = 10). The detected factors include p62 and HO-1, GCLM and Prdx1, as well as NQO1 and SLC7A11.

As shown in Figure 8, PAP-1 markedly regulated the protein expression of multiple antioxidant factors in RAW264.7 cells, with results largely consistent with the observed mRNA levels. Among the classical Nrf2 target proteins, HO-1 expression was significantly upregulated at 200 μg/mL and 50 μg/mL PAP-1 (*p* < 0.01); NQO1 was strongly elevated at 50 μg/mL (*p* < 0.01); and GCLM was significantly increased at 200 μg/mL (*p* < 0.01) and moderately elevated at 100 μg/mL (*p* < 0.05). Regarding other related factors, 200 μg/mL PAP-1 markedly enhanced p62 protein expression (*p* < 0.01), while 100 μg/mL PAP-1 significantly increased Prdx1 protein levels (*p* < 0.05). In addition, SLC7A11 expression was significantly upregulated at both 400 μg/mL and 200 μg/mL PAP-1 (*p* < 0.05), further supporting the antioxidative potential of PAP-1.

## 4. Discussion

This study systematically evaluated the effects of PAP-1 on the antioxidant capacity of mice and RAW264.7 cells. The results demonstrated that PAP-1 enhanced antioxidant enzyme activities and suppressed the production of oxidative stress-related biomarkers, thereby effectively strengthening the antioxidant defense systems in both cells and mice. In our group’s previous investigations, PAP-1 was found to markedly enhance the activity of antioxidant enzymes in virus-infected murine RAW264.7 macrophages [14]. To elucidate its intrinsic antioxidant properties under non-stressful physiological conditions, transcriptomic sequencing was performed on RAW264.7 cells treated with 200 μg/mL PAP-1 for 12 h. The enrichment analysis revealed 771 differentially expressed genes, of which 424 mRNAs were significantly upregulated and 347 mRNAs were significantly downregulated. Notably, several of these differentially expressed mRNAs were associated with molecular functions such as antioxidant activity and oxidoreductase activity, suggesting their potential involvement in antioxidant effects through immune- and oxidative stress-related pathways. Similarly, Li et al. [21], in their investigation of the immunomodulatory properties and molecular mechanisms of mung bean peel polysaccharides (MBP) in RAW264.7 macrophages, demonstrated that MBP enhanced macrophage phagocytic activity, promoted intracellular ROS production, and facilitated NO and cytokine release. Transcriptome sequencing identified 927 differentially expressed genes between the control and MBP-treated groups (196 upregulated, 731 downregulated), with KEGG enrichment analysis highlighting functional associations with Toll-like receptor 4 and NF-κB signaling pathways. In parallel, Yi et al. [22] examined the antioxidant activity of areca nut polyphenols (ANP) in LPS-stimulated RAW264.7 macrophages, revealing that ANP reduced ROS levels while upregulating Nrf2 and HO-1 expression. RNA-Seq analysis further indicated that 160 μg/mL ANP downregulated cancer-related pathways and gene transcription, whereas 320 μg/mL ANP suppressed inflammatory and viral infection pathways. The transcriptomic findings of the present study are consistent with these prior results, thereby providing the basis for further exploration of the antioxidant effects of PAP-1 both in vitro and in vivo.

Excessive production of MDA, MPO, XOD, and ROS in mice can trigger progressive inflammation, thereby inducing oxidative stress [23]. The endogenous antioxidant defense system of animals can be categorized into enzymatic and non-enzymatic components. The enzymatic system, primarily comprising superoxide dismutase (SOD), catalase (CAT), glutathione peroxidase (GSH-Px), and other endogenous antioxidant enzymes, represents the first line of defense against oxidative damage [24]. Antioxidant enzymes are regarded as critical mediators in alleviating oxidative stress, with SOD, CAT, and GSH-Px playing pivotal roles in neutralizing free radical-induced injury [25]. Specifically, GSH-Px catalyzes the reduction of superoxide radicals to hydrogen peroxide, while CAT subsequently decomposes hydrogen peroxide into water and oxygen, thereby attenuating oxidative damage [26]. The present study demonstrated that PAP-1 exerts antioxidant effects by elevating antioxidant substance levels and enhancing the activities of key antioxidant enzymes (SOD, CAT, GSH-Px), while simultaneously reducing pro-inflammatory factors (MDA, MPO, XOD), with consistent trends observed in both in vivo and in vitro experiments. Comparable antioxidative and anti-inflammatory effects have also been validated in other naturally derived polysaccharides. For instance, rice bran polysaccharides, when administered by gavage, significantly reduced oxidative stress products such as MDA in mouse serum, liver, and spleen, while enhancing the activities of antioxidant enzymes including SOD and CAT [27]. Ginkgo biloba polysaccharides exhibited broader anti-inflammatory and antioxidant activities, not only reducing serum inflammatory factor levels in alopecia areata mice but also markedly inhibiting the expression of key inflammatory signaling molecules such as p-p65, p-IκB, TNF-α, and IL-1β in human umbilical vein endothelial cells in vitro [28]. Similarly, Lentinula edodes polysaccharides have been shown to suppress the excessive expression of pro-inflammatory cytokines-including TNF-α, IL-6, IL-1β, and IFN-γ-in mouse colonic tissue, thereby mitigating inflammatory responses. Taken together, these findings suggest that polysaccharides from diverse natural sources share a common capacity to enhance antioxidant enzyme activity while suppressing pro-inflammatory mediators, a pattern that closely aligns with the antioxidant and anti-inflammatory properties of PAP-1 revealed in the present study.

Heme oxygenase-1 (HO-1), the rate-limiting enzyme in heme catabolism, plays a critical cytoprotective role. Under oxidative stress, HO-1 expression is subject to feedback regulation [29]; consequently, elevated levels of HO-1 are frequently observed in various pathological and oxidative stress conditions. NAD(P)H: quinone oxidoreductase 1 (NQO1) reduces quinone reactivity through dismutation reactions, thereby limiting the formation of reactive oxygen species (ROS) [30]. For example, in a rat model of severe acute pancreatitis, cinobufagin exhibited potent antioxidant effects by inhibiting the Keap1-Nrf2 interaction and promoting HO-1 expression [31]. Similarly, Lee et al. [32] reported that in an oxidative stress model of rat exocrine pancreatic cells (AR42J) induced by LPS and ethanol, lycopene prevented pancreatic inflammation by activating Nrf2, thereby upregulating NQO1 and HO-1 and suppressing ROS-mediated IL-6 expression in acinar cells. In collagen-induced arthritic mice, a sinomenine derivative was shown to activate Nrf2, leading to increased HO-1 and NQO1 expression, inhibition of osteoclast differentiation, and attenuation of joint inflammation. Moreover, this compound reduced the release of pro-inflammatory cytokines such as TNF-α and IL-17 and enhanced antioxidant enzyme activity by suppressing the MAPK and NF-κB pathways [33]. In the present study, hematoxylin-eosin staining of mouse spleen, thymus, and lung tissues revealed that PAP-1 attenuated oxidative tissue injury in these organs. Furthermore, immunohistochemical staining demonstrated increased expression of NQO1 and HO-1 in the spleen and thymus following PAP-1 treatment, findings consistent with the aforementioned studies, thereby indicating that PAP-1 exerts significant antioxidant effects in vivo.

SQSTM1/p62 (sequestosome-1) is a ubiquitin-binding protein implicated in cellular signaling, oxidative stress, and autophagy [34]. Within the p62-Keap1-Nrf2 axis, p62 functions as a key regulator of Nrf2 activation and also serves as a signaling hub for diverse cellular processes, including amino acid sensing and oxidative stress responses [35]. Metabolic stress enhances the expression and phosphorylation of SQSTM1/p62, and phosphorylation at Ser24 and Ser226 has been shown to activate the AMPK and NFE2L2/NRF2 pathways, thereby exerting synergistic antioxidant effects [36]. Previous studies demonstrated that treatment of 293T cells with varying concentrations of polysaccharides upregulated Nrf2 protein expression, as well as the downstream antioxidant proteins NQO1 and HO-1 [27]. Activation and increased expression of Nrf2 can markedly elevate HO-1 expression [37]. Moreover, evidence indicates that Lapachol (Lap) attenuates oxidative stress by reducing ROS generation via upregulation of NQO1 [38]. Consistent with these findings, the present study revealed that PAP-1 enhanced the protein expression of HO-1, NQO1, and p62, thereby exerting antioxidant effects. However, while p62 protein levels were elevated, p62 mRNA expression was reduced, showing partial divergence from transcriptomic data. This discrepancy may be attributable to the regulatory role of Nrf2 in p62 gene expression. Under conditions of selective autophagy, p62 mRNA levels may progressively increase, forming a positive feedback loop within the axis. Nonetheless, sustained Nrf2 activation can exert cytotoxic effects, leading to the establishment of negative feedback that ultimately suppresses p62 mRNA expression [39]. The cystine/glutamate antiporter solute carrier family 7 member 11 (SLC7A11/xCT) mediates cystine uptake, promotes glutathione synthesis, and sustains cell survival under oxidative stress, thereby exerting potent antioxidant functions [40]. In this study, PAP-1 at all tested concentrations significantly upregulated SLC7A11 mRNA expression (*p* < 0.01), while 400 μg/mL and 200 μg/mL PAP-1 also markedly increased SLC7A11 protein expression (*p* < 0.05), highlighting its role in modulating systemic antioxidant defense. Peroxiredoxins (Prdxs) constitute a highly conserved family of peroxidases [41]. Among them, Prdx1 exhibits peroxidase activity by reducing intracellular hydrogen peroxide (H_2_O_2_) levels and thereby regulating hydroxyl radical concentrations [42]. In the present study, Prdx1 protein expression was relatively low, which may be explained by the catalytic mechanism of Prdxs. During catalysis, the peroxidatic cysteine is oxidized by peroxides to a sulfenic acid intermediate, subsequently forming a disulfide bond with the resolving cysteine of the other subunit in the homodimer. This disulfide is then reduced by the thioredoxin system to regenerate the active thiol form. However, irreversible hyperoxidation of the catalytic cysteine to sulfinic or sulfonic acid leads to Prdx1 inactivation and degradation, accounting for its reduced activity [43]. It is noteworthy that the antioxidant activity of PAP-1 did not exhibit a strictly linear relationship. In certain experiments, the biological effects observed in the high-dose groups tended to plateau or fluctuate slightly, indicating a nonlinear trend. Such phenomena are relatively common among natural polysaccharide compounds and may be attributed to factors such as their complex molecular structures, receptor-binding affinities, and cellular uptake efficiencies [44]. Moreover, excessive activation of antioxidant or anti-inflammatory signaling pathways such as Nrf2 and NF-κB can trigger negative feedback regulation or signal saturation, resulting in a lack of proportional enhancement at higher doses [45]. These findings suggest that the antioxidant effects of PAP-1 may operate within an optimal effective dosage range rather than following a simple dose-dependent pattern. In summary, this study demonstrated that PAP-1 reduced the expression levels of inflammatory factors while markedly upregulating antioxidant enzymes and the expression of representative antioxidant genes and proteins, including HO-1, NQO1, GCLM, p62, Prdx1, and SLC7A11. The integrated alterations in these antioxidant-related parameters can be regarded as a multi-indicator evaluation model analogous to an “oxidation index,” providing a more systematic and accurate reflection of the overall antioxidant efficacy of PAP-1.

## 5. Conclusions

This study systematically evaluated the antioxidant effects of *Arthrospira platensis* polysaccharide fraction 1 (PAP-1) both in vitro and in vivo. The results demonstrated that PAP-1 markedly enhanced the antioxidant capacity of cells and murine tissues, while upregulating the expression of classical antioxidant genes, including HO-1, NQO1, GCLM, p62, Prdx1, and SLC7A11. These findings not only elucidate the molecular mechanisms underlying PAP-1 activity but also provide a theoretical basis for its potential development as a bioactive component of functional foods or as a candidate therapeutic intervention. Nevertheless, this study presents certain limitations. The current experiments primarily examined the antioxidant capacity of PAP-1 under physiological conditions, without validation in oxidative stress models such as H_2_O_2_ or UVB exposure. Moreover, although transcriptomic and biochemical analyses identified several genes associated with antioxidant activity, the direct molecular targets and binding mechanisms of PAP-1 remain to be clarified. Future studies integrating different treatment durations and pharmacokinetic assessments are warranted to provide a more comprehensive understanding of its biological functions. It is noteworthy that oxidative stress plays a pivotal role in the onset and progression of numerous diseases, including neurodegenerative disorders (such as Alzheimer’s and Parkinson’s diseases), cardiovascular diseases, metabolic syndromes, and inflammation-related pathologies. Therefore, as a natural polysaccharide with promising antioxidant potential, PAP-1 may hold future promise as a functional food ingredient or an adjuvant therapeutic candidate for these pathological conditions.

## Data Availability

The data are included in the article and Supplementary Materials.

## Supplementary Materials

The following supporting information can be downloaded at: https://www.mdpi.com/article/10.3390/antiox14111358/s1, Table S1. Grouping and Treatment of Mice; Table S2. Primer Sequences Used for RT-qPCR; Figure S1. GO/KEGG enrichment analysis of differentially expressed genes (means ± SD, *n* = 3).
