# Supplementary material for: In Vivo and In Vitro Antioxidant Effects of Arthrospira platensis Polysaccharide Component 1 (PAP-1)
Source: Antioxidants (Basel). 2025 Nov 13;14(11):1358. doi: 10.3390/antiox14111358 (PMC12649529; doi:10.3390/antiox14111358)
Supplement: Supplementary file 1 [file antioxidants-14-01358-s001.zip › antioxidants-3954343-supplementary.pdf]

antioxidants

MDPI

Article

In Vivo and In Vitro Antioxidant Effects of *Arthrospira platensis* Polysaccharide Component 1 (PAP-1)

Haifeng Yuan, Yuheng Wei, Zhaoyuan He, Xinrui Wang, Xiaoli Yu, Qiuhua Wang, Meiling Yu, Tingjun Hu *

College of Animal Science and Technology, Guangxi University, Nanning 530004, China; 2318302040@st.gxu.edu.cn (H. Y.); weiyuheng@st.gxu.edu.cn (Y. H.); z294he@uwaterloo.ca (Z. H.); 2018393054@st.gxu.edu.cn (X. W.); 2318393087@st.gxu.edu.cn (X. Y.); qiuhuawang@gxu.edu.cn (Q. W.); yumeiling@gxu.edu.cn (M. Y.)

* Correspondence: tingjunhu@gxu.edu.cn; Tel.: +86-771-3235635; Fax: +86-771-3270149

Keywords: *Arthrospira platensis*; polysaccharide component (PAP-1); antioxidant behaviour; oxidative stress; RNA-seq; *in vivo* study; cellular study

Supplementary Materials

Reagents

Dulbecco's Modified Eagle Medium (DMEM) was purchased from Gibco (USA); phosphate-buffered saline (PBS, powder form) was obtained from Beijing Solarbio Science & Technology Co., Ltd., (Beijing, China); fetal bovine serum (FBS) was provided by Wisent Biotechnology (Nanjing) Co., Ltd., (Nanjing, China); Trizol reagent and RNA extraction kits were purchased from Takara Biotechnology (Beijing) Co., Ltd., (Beijing, China); Assay kits for NO (Batch No.: 20220610), iNOS (Batch No.: 20220610), MDA (Batch No.: 20220610), SOD (Batch No.: 20220610), CAT (Batch No.: 20220620), MPO (Batch No.: 20220618), XOD (Batch No.: 20220610), GSH-Px (Batch No.: 20220617), and total protein quantification using the BCA method (Batch No.: 20220606) were all obtained from Nanjing Jiancheng Bioengineering Institute., (Nanjing, China); The general SP (Mouse/Rabbit IgG)-POD Kit (Batch No.: 20230726) was acquired from Beijing Solarbio Science & Technology Co., Ltd., (Beijing, China); Recombinant Anti-Heme Oxygenase 1 (HO-1) antibody and recombinant Anti-NQO1 antibody were purchased from Abcam (Shanghai) Trading Co., Ltd., (Shanghai, China); NO (Batch No.: 20200812), iNOS (Batch No.: 20200811), MDA (Batch No.: 20200812), SOD (Batch No.: 20200811), CAT (Batch No.: 20200813), MPO (Batch No.: 20200812), XOD (Batch No.: 20200812), GSH-Px (Batch No.: 20200807), and BCA protein quantification kit (Batch No.: 20200622) were also supplied by Nanjing Jiancheng Bioengineering Institute., (Nanjing, China); The 5× All-In-One RT MasterMix with AccuRT for reverse transcription and EvaGreen 2× qPCR MasterMix-No Dye for real-time quantitative PCR were purchased from abm (Canada). Recombinant Anti-HO-1 antibody, recombinant Anti-NQO1 antibody, and Goat Anti-Mouse IgG H&L (HRP) were all obtained from Abcam (UK). Antibodies against iNOS and β-actin (13E5) Rabbit mAb were sourced from Cell Signaling Technology (CST, USA). Antibodies for SQSTM1/p62, Peroxiredoxin 1 (Prdx1), GCLM, and SLC7A11/xCT were purchased from

 https://doi.org/10.3390/antiox14111358

Affinity Biosciences (USA). The Direct-load™ pre-stained protein molecular weight marker (10–180 kDa) was obtained from GenStar (USA). RIPA lysis buffer (strong) was supplied by Beyotime Biotechnology (China). Solutions including 30% acrylamide gel solution, 1 M Tris-HCl buffer (pH 6.8), 1.5 M Tris-HCl buffer (pH 8.8), 20× TBST buffer, glycine, sodium dodecyl sulfate (SDS), Tris(hydroxymethyl)aminomethane (Tris), and 5× protein loading buffer were all purchased from Beijing Solarbio Science & Technology Co., Ltd., (Beijing, China); The NcmECL Ultra-sensitive chemiluminescent substrate was obtained from Suzhou NewSaier Biotech Co., Ltd., (Suzhou, China); All other reagents used were of analytical grade.

Preparation of PAP-1 Solution

Arthrospira platensis powder was supplied by Beihai Shengbada Biotechnology Co., Ltd., (Beihai, China) under the supervision of the Institute of Hydrobiology, Chinese Academy of Sciences. PAP was extracted via enzymatic hydrolysis and aqueous extraction, followed by successive purification through DEAE-52 cellulose ion-exchange chromatography and Sephacryl S-200 gel filtration chromatography to obtain PAP-1. Accurately weighed PAP-1 was dissolved in DMEM culture medium and serially diluted to the desired concentrations (400 μg/mL, 200 μg/mL, 100 μg/mL, and 50 μg/mL), then sterilized by filtration through a 0.22 μm membrane filter and freshly prepared prior to use. For in vivo administration, precise amounts of PAP-1 powder were dissolved in sterile physiological saline (SPS) to prepare solutions at varying doses (400 mg/kg·BW, 200 mg/kg·BW, 100 mg/kg·BW, and 50 mg/kg·BW), freshly prepared immediately before use.

Animal Grouping and Treatment

Sixty specific pathogen-free (SPF) Kunming mice, evenly divided by sex, weighing 18-22 g and aged 6 weeks, were obtained from the Experimental Animal Center of Guangxi Medical University. Approval was obtained from the Ethics Committee of Guangxi University. The procedures used in this study adhere to the tenets of the Declaration of Helsinki. The study was conducted in accordance with the local legislation and institutional requirements. Before the experiments commenced, the mice were acclimated for seven days under controlled environmental conditions (22 ± 2 °C) with ad libitum access to food and water. To minimize sex-related bias, an equal number of male and female mice were included in each group. The animals were first stratified by sex, after which simple randomization was performed within each sex group using a random number generator. A total of sixty mice were thus allocated into six groups, with ten mice per group, comprising equal numbers of males and females. Following acclimation, the mice were orally administered the six prepared solutions according to Table S1. The vitamin C (Vc) group received sterile physiological saline (SPS) prepared at 400 mg/kg·BW. Treatments were administered daily for seven consecutive days. On day seven, mice were euthanized by cervical dislocation; blood was collected via orbital puncture, and thymus, spleen, and lung tissues were harvested. Serum was isolated from blood samples for the assessment of redox-related biomarkers. Tissue samples were fixed in 4% paraformaldehyde for subsequent hematoxylin and eosin (H&E) staining and immunohistochemistry (IHC) analyses.

Table S1. Grouping and Treatment of Mice

Group	Treatment Description	Dose (mg/kg·BW)	Number of Mice (n)
Control	SPS (Control)	/	10 (♂5, ♀5)
Vc	Vc (positive control), dissolved in SPS	400	10 (♂5, ♀5)
50-PAP-1	PAP-1 dissolved in SPS	50	10 (♂5, ♀5)
100-PAP-1	PAP-1 dissolved in SPS	100	10 (♂5, ♀5)
200-PAP-1	PAP-1 dissolved in SPS	200	10 (♂5, ♀5)
400-PAP-1	PAP-1 dissolved in SPS	400	10 (♂5, ♀5)

Grouping and Treatment of RAW264.7 Cells

The murine macrophage cell line RAW264.7 was obtained from the Cell Bank of Wuhan University and cryopreserved by the Laboratory of Veterinary Pharmacology, College of Animal Science and Technology, Guangxi University. Cells were cultured in DMEM supplemented with 10% fetal bovine serum (FBS; Gibco), 100 IU/mL penicillin, and 100 μg/mL streptomycin, and maintained at 37°C in a humidified incubator with 5% CO_2. The experimental design included six groups: a control group, a vitamin C (Vc) group, and four PAP-1 treatment groups at concentrations of 400 μg/mL, 200 μg/mL, 100 μg/mL, and 50 μg/mL. When RAW264.7 cells in the logarithmic growth phase reached 70-80% confluence in 6-well plates, the culture medium was discarded, and the cells were washed three times with PBS. Sterile-filtered PAP-1 solutions at the indicated concentrations were added to the respective treatment groups. The Vc group received 400 μg/mL of sterile-filtered vitamin C solution, while the control group received an equal volume of complete culture medium without any treatment. All groups were incubated for an additional 12 h under standard conditions (37 °C, 5% CO_2), after which the cells were harvested for *in vitro* antioxidant activity assays.

Parameter for RT-qPCR Analysis

Total RNA was extracted strictly in accordance with the instructions provided in the RNA purification kit. A 1 μL aliquot of RNA was used to assess purity using a micro-spectrophotometer, while the remaining RNA was reverse-transcribed into cDNA using the abm reverse transcription kit, following the manufacturer's protocol. The qPCR reaction mixture (20 μL total volume) was prepared as follows: 10 μL BlasTaq™ 2× qPCR MasterMix, 0.5 μL forward primer, 0.5 μL reverse primer, 1 μL cDNA template, and 8 μL nuclease-free water. The amplification protocol consisted of an initial denaturation at 95 °C for 3 minutes, followed by 40 cycles of 95 °C for 15 seconds and 60 °C for 1 minute. Primers for HO-1, NQO1, GCLM, p62, Prdx1, and SLC7A11 were synthesized by Nanning Genis Biotech Co., Ltd., and their sequences (5′to3′) are listed in Table S2. β-actin served as the internal reference gene, and relative mRNA expression levels were calculated using the $2^{\wedge-\Delta\Delta Ct}$ method.

Table S2. Primer Sequences Used for RT-qPCR

Target Gene	Sequence (5′-3′)	Accession	PCR Production (bp)
GADPH	F: GACGGCCAGGTCATCACTATTG<br>R: AGTTTCATGGATGCCACAGGAT	XM_021091114.1	129
GCLM	F: GCCACCAGATTTGACTGCCTTT	NM_008129.4	119

	R: CAGGGATGCTTTCTTGAAGAGCTT		
Prdx1	F: AGTCCAGGCCTTCCAGTTCACT<br>R: GGCTTGATGGTATCACTGCCAG	XM_051171941.1	81
HO-1	F: ACATCGACAGCCCCACCAAGTTCAA<br>R: CTGACGAAGTGACGCCATCTGTGAG	NM_010442.2	203
NQO1	F: CATTCTGAAAGGCTGGTTTGA<br>R: TTTCTTCCATCCTTCCAGGAT	XM_029480809.1	298
P62	F: ATGGAGTCGGGAAACTGCTC<br>R: AACCCATGGACAGCATCTGG	NM_001290769.1	127
Slc7a11	F: GGCACCGTCATCGGATCAG<br>R: CTCCACAGGCAGACCAGAAAA	XM_021158042.1	100

Differential GO/KEGG Analysis

To further elucidate the functional implications of PAP-1 treatment on differentially expressed genes, GO and KEGG enrichment analyses were conducted. The hierarchical distribution of GO annotations (Figure S1A) revealed that differentially expressed mRNAs were primarily enriched in biological processes (including cellular processes, biological regulation, metabolic processes, responses to stimuli, signal transduction, and immune processes), cellular components (such as cellular compartments, organelles, cell membranes, and macromolecular complexes), and molecular functions (predominantly protein binding). Specifically, within the biological process category, a total of 3,032 transcripts were identified, with significant enrichment in GO:0048519 negative regulation of biological process and GO:1901700 response to oxygen-containing compound, indicating a close association with the regulation of oxidative stress (Figure S1B). In the molecular function category, 2,082 transcripts were significantly enriched in protein binding (GO:0005515), antioxidant activity (GO:0016209), binding (GO:0005488), and oxidoreductase activity, acting on peroxide as acceptor (GO:0016684) (Figure S1C). Within the cellular component category, 6,434 transcripts were mainly distributed across the cytoplasmic part (GO:0044444), plasma membrane part (GO:0044459), and whole membrane (GO:0098805), followed by the plasma membrane (GO:0005886) (Figure S1D).

By annotating all transcripts from the control and 200 μg/mL PAP-1 groups to the KEGG database, differential expression analysis was performed on the annotated pathways. The results (Figure S1E) revealed 1,046 differentially expressed genes signi ficantly enriched in 41 signaling pathways, including immune system-related pathways (involving 73 differentially expressed genes). A total of 303 transcripts were mapped to KEGG pathways, with predominant enrichment in classical antioxidant and inflammation-regulatory signaling cascades such as FoxO, IL-17, NF-κB, and Toll-like receptor pathways (Figure S1F). These findings suggest that PAP-1 may exert its antioxidative and anti-inflammatory effects through coordinated regulation of multiple signaling networks.

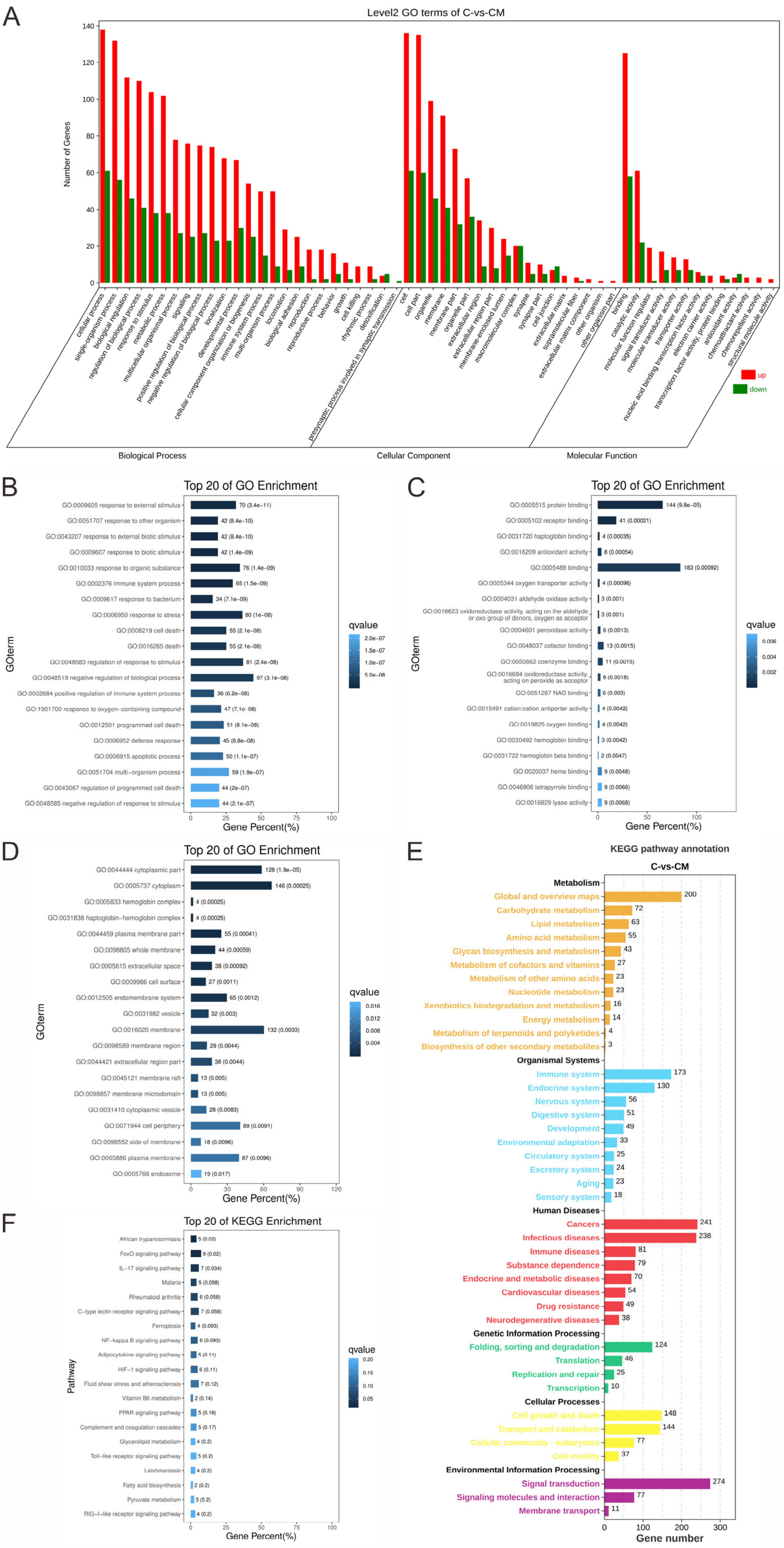
A
Level2 GO terms of C-vs-CM
Number of Genes
up
down
Biological Process
Cellular Component
Molecular Function
cellular process
single-organism process
biological regulation
regulation of biological process
response to stimulus
metabolic process
multicellular organismal process
signaling
positive regulation of biological process
negative regulation of biological process
localization
developmental process
cellular component organization or biogenesis
immune system process
multi-organism process
locomotion
biological adhesion
reproduction
reproductive process
behavior
growth
cell killing
rhythmic process
detoxification
presynaptic process involved in synaptic transmission
cell
cell part
organelle
membrane
membrane part
organelle part
extracellular region
extracellular region part
membrane-enclosed lumen
macromolecular complex
synapse
synapse part
cell junction
extracellular matrix
supramolecular fiber
extracellular matrix component
other organism
other organism part
binding
catalytic activity
molecular function regulator
signal transducer activity
molecular transducer activity
transporter activity
nucleic acid binding transcription factor activity
electron carrier activity
transcription factor activity, protein binding
antioxidant activity
chemoattractant activity
chemorepellent activity
structural molecule activity
B
Top 20 of GO Enrichment
GOterm
qvalue
Gene Percent(%)
GO:0009605 response to external stimulus 70 (3.4e−11)
GO:0051707 response to other organism 42 (8.4e−10)
GO:0043207 response to external biotic stimulus 42 (8.4e−10)
GO:0009607 response to biotic stimulus 42 (1.4e−09)
GO:0010033 response to organic substance 76 (1.4e−09)
GO:0002376 immune system process 65 (1.5e−09)
GO:0009617 response to bacterium 34 (7.1e−09)
GO:0006950 response to stress 80 (1.1e−08)
GO:0008219 cell death 55 (2.1e−08)
GO:0016265 death 55 (2.1e−08)
GO:0048583 regulation of response to stimulus 81 (2.4e−08)
GO:0048519 negative regulation of biological process 97 (3.1e−08)
GO:0002684 positive regulation of immune system process 36 (6.2e−08)
GO:1901700 response to oxygen−containing compound 47 (7.1e−08)
GO:0012501 programmed cell death 51 (8.1e−08)
GO:0006952 defense response 45 (8.8e−08)
GO:0006915 apoptotic process 50 (1.1e−07)
GO:0051704 multi−organism process 59 (1.9e−07)
GO:0043067 regulation of programmed cell death 44 (2e−07)
GO:0048585 negative regulation of response to stimulus 44 (2.1e−07)
C
Top 20 of GO Enrichment
GOterm
qvalue
Gene Percent(%)
GO:0005515 protein binding 144 (9.8e−05)
GO:0005102 receptor binding 41 (0.00021)
GO:0031720 haptoglobin binding 4 (0.00035)
GO:0016209 antioxidant activity 8 (0.00054)
GO:0005488 binding 183 (0.00092)
GO:0005344 oxygen transporter activity 4 (0.00096)
GO:0004031 aldehyde oxidase activity 3 (0.001)
GO:0016623 oxidoreductase activity, acting on the aldehyde or oxo group of donors, oxygen as acceptor 3 (0.001)
GO:0004601 peroxidase activity 6 (0.0013)
GO:0048037 cofactor binding 13 (0.0015)
GO:0050662 coenzyme binding 11 (0.0015)
GO:0016684 oxidoreductase activity, acting on peroxide as acceptor 6 (0.0018)
GO:0051287 NAD binding 6 (0.003)
GO:0015491 cation:cation antiporter activity 4 (0.0042)
GO:0019825 oxygen binding 4 (0.0042)
GO:0030492 hemoglobin binding 3 (0.0042)
GO:0031722 hemoglobin beta binding 2 (0.0047)
GO:0020037 heme binding 9 (0.0048)
GO:0046906 tetrapyrrole binding 9 (0.0066)
GO:0016829 lyase activity 9 (0.0068)
D
Top 20 of GO Enrichment
GOterm
qvalue
Gene Percent(%)
GO:0044444 cytoplasmic part 128 (1.9e−05)
GO:0005737 cytoplasm 146 (0.00025)
GO:0005833 hemoglobin complex 4 (0.00025)
GO:0031838 haptoglobin−hemoglobin complex 4 (0.00025)
GO:0044459 plasma membrane part 55 (0.00041)
GO:0098805 whole membrane 44 (0.00059)
GO:0005615 extracellular space 38 (0.00092)
GO:0009986 cell surface 27 (0.0011)
GO:0012505 endomembrane system 65 (0.0012)
GO:0031982 vesicle 32 (0.003)
GO:0016020 membrane 132 (0.0033)
GO:0098589 membrane region 29 (0.0044)
GO:0044421 extracellular region part 38 (0.0044)
GO:0045121 membrane raft 13 (0.005)
GO:0098857 membrane microdomain 13 (0.005)
GO:0031410 cytoplasmic vesicle 28 (0.0083)
GO:0071944 cell periphery 89 (0.0091)
GO:0098552 side of membrane 18 (0.0096)
GO:0005886 plasma membrane 87 (0.0096)
GO:0005768 endosome 19 (0.017)
E
KEGG pathway annotation
C-vs-CM
Metabolism
Global and overview maps 200
Carbohydrate metabolism 72
Lipid metabolism 63
Amino acid metabolism 55
Glycan biosynthesis and metabolism 43
Metabolism of cofactors and vitamins 27
Metabolism of other amino acids 23
Nucleotide metabolism 23
Xenobiotics biodegradation and metabolism 16
Energy metabolism 14
Metabolism of terpenoids and polyketides 4
Biosynthesis of other secondary metabolites 3
Organismal Systems
Immune system 173
Endocrine system 130
Nervous system 56
Digestive system 51
Development 49
Environmental adaptation 33
Circulatory system 25
Excretory system 24
Aging 23
Sensory system 18
Human Diseases
Cancers 241
Infectious diseases 238
Immune diseases 81
Substance dependence 79
Endocrine and metabolic diseases 70
Cardiovascular diseases 54
Drug resistance 49
Neurodegenerative diseases 38
Genetic Information Processing
Folding, sorting and degradation 124
Translation 46
Replication and repair 25
Transcription 10
Cellular Processes
Cell growth and death 148
Transport and catabolism 144
Cellular community - eukaryotes 77
Cell motility 37
Environmental Information Processing
Signal transduction 274
Signaling molecules and interaction 77
Membrane transport 11
Gene number
F
Top 20 of KEGG Enrichment
Pathway
qvalue
Gene Percent(%)
African trypanosomiasis 5 (0.02)
FoxO signaling pathway 9 (0.02)
IL−17 signaling pathway 7 (0.034)
Malaria 5 (0.058)
Rheumatoid arthritis 6 (0.058)
C−type lectin receptor signaling pathway 7 (0.058)
Ferroptosis 4 (0.093)
NF−kappa B signaling pathway 6 (0.093)
Adipocytokine signaling pathway 5 (0.11)
HIF−1 signaling pathway 6 (0.11)
Fluid shear stress and atherosclerosis 7 (0.12)
Vitamin B6 metabolism 2 (0.14)
PPAR signaling pathway 5 (0.16)
Complement and coagulation cascades 5 (0.17)
Glycerolipid metabolism 4 (0.2)
Toll−like receptor signaling pathway 5 (0.2)
Leishmaniasis 4 (0.2)
Fatty acid biosynthesis 2 (0.2)
Pyruvate metabolism 3 (0.2)
RIG−I−like receptor signaling pathway 4 (0.2)

Figure S1. GO/KEGG enrichment analysis of differentially expressed genes (means ± SD, $n = 3$). (**A**) Hierarchical distribution of GO annotations. (**B**) Bar chart of GO enrichment analysis for biological processes. (**C**) Bar chart of GO enrichment analysis for molecular functions. (**D**) Bar chart of GO enrichment analysis for cellular components. (**E**) Hierarchical (category) distribution of KEGG annotations. (**F**) Bar chart of KEGG pathway enrichment analysis.
